# Replacement of neuraminidase inhibitor‐susceptible influenza A(H1N1) with resistant phenotype in 2008 and circulation of susceptible influenza A and B viruses during 2009‐2013, South Africa

**DOI:** 10.1111/irv.12611

**Published:** 2018-10-31

**Authors:** Florette K. Treurnicht, Amelia Buys, Stefano Tempia, Mpho Seleka, Adam L. Cohen, Sibongile Walaza, Allison J. Glass, Inéz Rossouw, Johanna McAnerney, Lucille Blumberg, Cheryl Cohen, Marietjie Venter

**Affiliations:** ^1^ Centre for Respiratory Diseases and Meningitis National Institute for Communicable Diseases of the National Health Laboratory Service Johannesburg South Africa; ^2^ Influenza Division Centers for Disease Control and Prevention Atlanta Georgia; ^3^ Influenza Program Centers for Disease Control and Prevention Pretoria South Africa; ^4^ Department of Immunization, Vaccines and Biologicals Global Immunization Monitoring and Surveillance, Expanded Programme on Immunization World Health Organization Geneva Switzerland; ^5^ Faculty of Health Sciences School of Public Health University of the Witwatersrand Johannesburg South Africa; ^6^ Department of Molecular Pathology Lancet Laboratories Johannesburg South Africa; ^7^ PathCare Laboratories PathCare Park Cape Town South Africa; ^8^ Division of Public Health Surveillance and Response National Institute of Communicable Diseases Johannesburg South Africa; ^9^ Department of Medical Virology Emerging Arbo‐and Respiratory Virus Program University of Pretoria Pretoria South Africa; ^10^ Tshwane Academic Division National Health Laboratory Service Pretoria South Africa

**Keywords:** influenza, oseltamivir, South Africa, susceptibility

## Abstract

**Background:**

Data on the susceptibility of influenza viruses from South Africa to neuraminidase inhibitors (NAIs) are scarce, and no extensive analysis was done.

**Objectives:**

We aimed to determine oseltamivir and zanamivir susceptibility of influenza A and B virus neuraminidases (NAs), 2007‐2013, South Africa.

**Patients/Methods:**

We enrolled participants through national influenza‐like illness surveillance, 2007‐2013. Influenza diagnosis was by virus isolation and quantitative polymerase chain reaction (qPCR). Drug susceptibility was determined by chemiluminescence‐based NA‐STAR/NA‐XTD assay. Sanger sequencing was used to determine molecular markers of NAI resistance.

**Results:**

Forty percent (6341/15 985) of participants were positive for influenza viruses using virus isolation (2007‐2009) and qPCR (2009‐2013) methods. A total of 1236/6341 (19.5%) virus isolates were generated of which 307/1236 (25%) were tested for drug susceptibility. During 2007‐2008, the median 50% inhibitory concentration (IC_50_) of oseltamivir for seasonal influenza A(H1N1) increased from of 0.08 nmol/L (range 0.01‐3.60) in 2007 to 73 nmol/L (range 1.56‐305 nmol/L) in 2008. Influenza A isolates from 2009 to 2013 were susceptible to oseltamivir [A(H3N2) median IC_50_ = 0.05 nmol/L (range 0.01‐0.08); A(H1N1)pdm09 = 0.11 nmol/L (range 0.01‐0.78)] and zanamivir [A(H3N2) median IC_50_ = 0.56 nmol/L (range 0.47‐0.66); A(H1N1)pdm09 = 0.35 nmol/L (range 0.27‐0.533)]. Influenza B viruses were susceptible to both NAIs. NAI resistance‐associated substitutions H275Y, E119V, and R150K (N1 numbering) were not detected in influenza A viruses that circulated in 2009‐2013.

**Conclusions:**

We confirm replacement of NAI susceptible by resistant phenotype influenza A(H1N1) in 2008. Influenza A and B viruses (2009‐2013) remained susceptible to NAIs; therefore, these drugs are useful for treating influenza‐infected patients.

## 1 INTRODUCTION

Annually, influenza virus infections account for an estimated 3‐5 million cases globally, with 250 000‐500 000 deaths.^1^ During the 2009 pandemic, an estimated 200 million infections occurred globally and resulted in approximately 138 000 deaths (range 123 000‐155 000).^2^, ^3^ Following the emergence of adamantine‐resistant influenza A viruses, clinical treatment of influenza virus disease is mainly with neuraminidase inhibitors (NAIs): Both zanamivir and oseltamivir was approved in 1999 by the US Food and Drug Administration to treat seasonal influenza.^4^, ^5^ Oseltamivir is the most widely used due to ease of oral administration. Data from Australasia and South‐East Asia showed that influenza A viruses from 1998 through 2002 were overall more susceptible to the NAIs, oseltamivir, and zanamivir, than influenza B viruses.^6^ However, in 2008, oseltamivir resistance was reported at a frequency of 90% globally for seasonal influenza A(H1N1) viruses and was associated with histidine to tyrosine mutation at position 275 (H275Y, N1 numbering) of the NA.^7^, ^8^

During the 2008 influenza season, oseltamivir‐resistant influenza A(H1N1) viruses were also isolated from South African patients with influenza‐like illness. These influenza A(H1N1) virus isolates (n = 49) had the H275Y substitution and were confirmed to be phenotypically resistant to inhibition by oseltamivir.^9^

Neuraminidase inhibitor resistance associated with the H275Y mutation was reported at a frequency of 3% (169/5152) in influenza A(H1N1)pdm09 virus isolates received at the World Health Organization (WHO) collaborating centers (CCs) from various geographic regions, 2013‐2014.^7^, ^10^ Global NAI susceptibility surveillance data from WHO‐CCs for 2013‐2014 include less than 3% African data.^10^

Both oseltamivir and zanamivir are licenced in South Africa. Zanamivir is available since the early 2000s and oseltamivir since 2006.^11^, ^12^ Zanamivir is approved for treatment of children aged 7 years and older, whereas oseltamivir can be given to individuals of all ages.^13^, ^14^ Although generally thought not to be widely prescribed, limited reports are available on the use of NAIs in South Africa. Benefit of oseltamivir for both treatment of and prophylaxis against influenza‐associated respiratory illness in South African infants with low birthweight was reported.^15^ Influenza A(H1N1)pdm09 H275Y‐resistant phenotype viruses were reported in 1 of 54 (2%) of patients following 5‐day standard dose oseltamivir treatment.^16^

We aimed to determine oseltamivir and zanamivir susceptibility of influenza A and B virus NAs, 2007‐2013, South Africa, and to investigate amino acid polymorphisms in NA.

## 2 METHODS

### 2.1 Surveillance programs

A sentinel surveillance program for influenza‐like illness (ILI) (Viral Watch [VW] program) recruited outpatients with a measured fever ≥38°C and cough, headache, myalgia, or sore throat (of duration ≤10 days) during 2007‐2013, through medical practitioners in all nine provinces of South Africa.^17^

### 2.2 Study specimens

**Figure 1:**
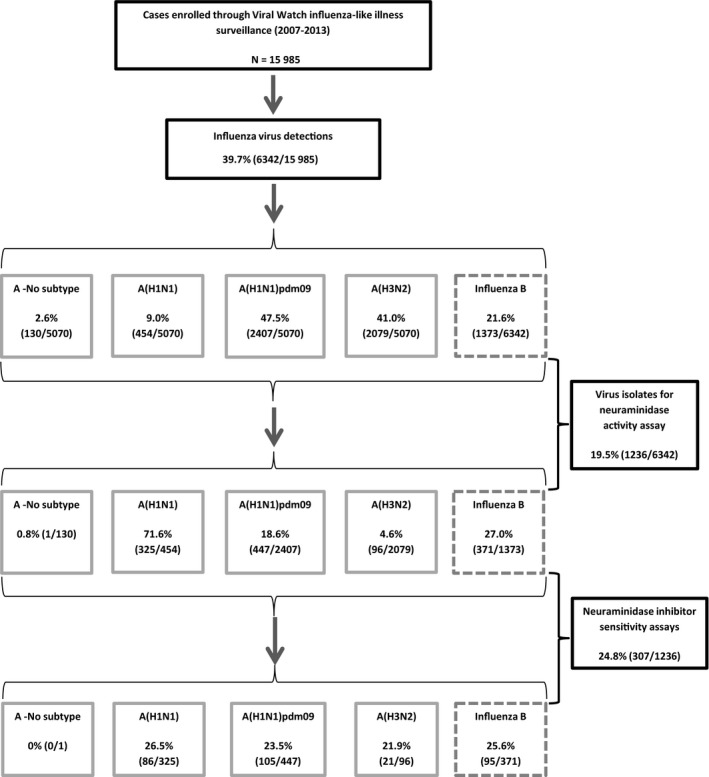
Flow diagram of influenza virus detections, virus isolates, and neuraminidase inhibitor susceptibility testing for cases enrolled through influenza‐like illness (Viral Watch) surveillance program, South Africa, 2007‐2013

Respiratory specimens collected included primarily nose and throat swabs collected at the time of diagnosis of the acute respiratory illness episode prior to the initiation of treatment. All upper respiratory tract specimens from patients enrolled from 2007 to 2013 (Figure 1) were collected in viral transport medium (Highveld Biological, Johannesburg, South Africa) or universal transport medium (Copan, Murrieta, CA, USA) as previously described.^18^, ^19^ Isolation of respiratory viruses including influenza A and B in Madin‐Darby canine kidney (MDCK) cell cultures was done for specimens submitted during 2007‐2009. From 2009, real‐time or quantitative reverse transcription‐polymerase chain reaction (qRT‐PCR) assays were introduced to test respiratory specimens by single or multiplex respiratory virus qPCR assays, which included diagnosis of influenza A and B viruses.^18^, ^20^, ^21^

### 2.3 Influenza virus detection and isolation

Before 2009, influenza virus isolation was performed using MDCK cells and immunofluorescence assays (IFAs) with monoclonal antibodies from Light Diagnostics^™^ (EMD Millipore Corp, Temecula, CA, USA) were used to confirm influenza A and B positive cultures. Briefly, MDCK cells were seeded in shell vials and maintained with Dulbecco's modified Eagle's medium (DMEM, Lonza, Allendale, NJ, USA) containing 4.5 g/L glucose, with l‐glutamine, 10% fetal bovine serum, 2 mmol/L l‐glutamine, and antibiotics. After inoculation with 250 microliter clinical specimen for 2 hours on washed serum‐free cell monolayers, the cultures were incubated with DMEM serum media at 37ºC and harvested 24‐73 hours later. Culture supernatant fluids from influenza virus cultures were stored at −70°C.^22^, ^23^ Influenza virus isolates (2007‐2009) were subtyped serologically by hemagglutination inhibition (HAI) and subtype‐specific antisera.^23^, ^24^, ^25^

Influenza viruses were detected by qRT‐PCR, and influenza A positives were subtyped using a qRT‐PCR assay from Centers for Disease Control and Prevention (CDC, Atlanta, GA, USA).^18^, ^20^, ^21^, ^26^ From 2009 to 2013, influenza‐positive specimens with qPCR with cycle threshold (*C*_t_) values <30 were selected for virus isolation.

In addition, we selected specimens for influenza virus isolation at the beginning, middle, and end of the South African influenza season (typically May through September).

### 2.4 Phenotypic drug resistance assays

#### 2.4.1 Neuraminidase inhibitor‐susceptible and neuraminidase inhibitor‐resistant reference virus isolates

Reference virus isolates (received from Dr L. Gubareva, CDC, GA, USA) were used as controls: (a) A/Washington/10/2008 [A(H1N1)], wild‐type strain, oseltamivir and zanamivir susceptible; (b) A/Florida/21/2008 [A(H1N1)], H275Y NA substitution, oseltamivir resistant and zanamivir susceptible; (c) A/Washington/01/2007 [A(H3N2)], wild‐type strain, oseltamivir and zanamivir susceptible; (d) A/Texas/12/2007 [A(H3N2)], E119V NA substitution, oseltamivir resistant and zanamivir susceptible; (e) B/Memphis/20/1996 (influenza B), wild‐type strain, oseltamivir and zanamivir susceptible; (f) B/Memphis/20/1996 (influenza B), R152K (N2 numbering) or R150K (N1 numbering); NA substitution, oseltamivir and zanamivir resistant; (g) A/California/07/2009 [A(H1N1)pdm09], wild‐type strain, oseltamivir and zanamivir susceptible; and (h) A/Texas/48/2009 [A(H1N1)pdm09], H275Y NA substitution, oseltamivir resistant and zanamivir susceptible.^7^ N1 numbering is used in manuscript.

#### 2.4.2 The NA‐STAR and NA‐XTD chemiluminescent assay

The NA‐STAR and NA‐XTD chemiluminescent assays (Applied Biosystems, Foster City, CA, USA) were used to determine the susceptibility of NA from influenza A and B virus isolates to oseltamivir and zanamivir. Virus culture supernatant fluid aliquots were retrieved and pre‐treated with 10% Triton X‐100 solution to inactivate virus.^27^ The NA inhibition assays were performed following the manufacturers’ protocols as described.^28^ Briefly, virus isolates were titrated to determine the signal‐to‐noise ratio of 40 as NA concentration for use in the inhibition assay. The final NAI concentrations ranging from 0.028 to 550 nanomolar (nmol/L) and the appropriate virus dilution (signal:noise = 40 or a minimum concentration of NA ≥40 000 relative light units [RLU]) were pre‐incubated for 30 min at room temperature in white flat‐bottom 96‐well plates (Microlite^™^2+, Thermo Fisher Scientific, Waltham, MA, USA). The chemiluminescence reaction was started with addition of NA‐STAR/NA‐XTD substrate followed by incubation for 15 min at room temperature. Following incubation, light emission was triggered by addition of accelerator buffer. Plates were read with the Glomax luminometer (Promega Inc, Madison, Wisconsin, USA).

The 50% inhibitory concentration (IC_50_) of oseltamivir and zanamivir was determined with the Robust Statistics (Robosage) MS Excel program add‐in from AMC Statistics (http://www.rsc.org/Membership/Networking/InterestGroups/Analytical/AMC/Software/RobustStatistics.asp). The quartiles and interquartile ranges (IQRs) of the antiviral concentration in log scale were obtained using box‐and‐whisker analyses and were subsequently back‐transformed to obtain final antiviral concentrations in nmol/L. Statistical cutoffs for identification of virus strains with reduced NAI susceptibility (or outliers) were defined as: *X*_0.75_ + 3IQR (with *X*_0.75_ = 75th percentile). Outliers were defined as viruses with IC_50_ values > cutoff and >10 times the median or mean IC_50_.^7^, ^28^ The NAIs oseltamivir (Tamiflu, the metabolized active form oseltamivir carboxylate was used in our assays) and zanamivir (Relenza) were obtained from Hoffmann‐La Roche Ltd (Basel, Switzerland) and GlaxoSmithKline (Stevenage, UK). Observed oseltamivir IC_50_ values reported for resistant phenotype reference viruses were as follows: seasonal A(H1N1) = 93.24 nmol/L, A(H1N1)pdm09 = 70.63 nmol/L, A(H3N2) = 5.24 nmol/L, and influenza B was 92.97 nmol/L for oseltamivir and 27.90 nmol/L for zanamivir with the NA‐XTD assay. Reference strains susceptible to oseltamivir and zanamivir had the following respective IC_50_ values: A(H1N1) = 0.13 and 0.35 nmol/L, A(H1N1)pdm09 = 0.10 and 0.42 nmol/L, A(H3N2) = 0.21 and 1.01 nmol/L, and influenza B = 0.68 and 2.58 nmol/L.^29^

### 2.5 Detection of drug resistance mutations and drift in influenza virus neuraminidase

The NA genes of influenza A and B strains from 2009 to 2013 were amplified and sequenced from original clinical specimens. Briefly, the universal influenza A and B primers (uni12 and Buni11) were used for complementary DNA (cDNA) preparation followed by nested PCR.^30^, ^31^ Sequencing was performed using the BigDye V3.1 chain terminator sequencing reaction ready mix (Applied Biosystems). Sequence chromatograms were assembled and verified using Sequencher^®^ version 5.4.6 DNA sequence analysis software (Gene Codes Corporation, Ann Arbor, MI, USA, http://www.genecodes.com ). Multiple sequence alignment and translation to deduced amino acid sequences were done with the bioedit software program.^32^ NA sequences of influenza A(H1N1)pdm09, A(H3N2), and influenza B viruses generated were screened for the presence of known molecular markers (N1 numbering) of NAI resistance that demonstrated clinical relevance in influenza A viruses of N1 (I223R, H275Y) or N2 (E119V/I, R293K, N295S) subtypes or influenza B viruses (R150K, D197E).^28^, ^33^, ^34^, ^35^, ^36^, ^37^ NAI resistance markers were also identified through surveillance or reverse genetics studies in N1 (E119V, D199G, I223K, N295S) or N2 (E119D, D151E/V, R224K, E276D, R371K) subtypes and influenza B (E117A/D/G/V, P139S, G140R, D197Y, R292K).^28^, ^34^, ^35^, ^36^, ^37^, ^38^, ^39^, ^40^, ^41^, ^42^

## 3 Results

### 3.1 Influenza viruses detected and isolated

A total of 15 985 specimens were submitted through VW surveillance from 2007 to 2013, of which 6342 (39.7%) specimens were positive for influenza. Of these, 5070 (79.9%) were influenza A (including 101 dual positives; A(H3N2)/A(H1N1)pdm09: 25, A(H3N2)/B: 47, A(H1N1)pdm09/B: 28, A/B: 1) and 1373 were influenza B (21.6%) (Figure 1, Table 1). Influenza A positives comprised of A(H1N1) 9.0% (454/5070), A(H1N1)pdm09 47.5% (2407/5070), A(H3N2) 41.0% (2079/5070), and A no subtype (subtype or serotype could not be determined) 2.6% (130/5070). Culture supernatant fluids from 1236 influenza virus isolates were tested for viral NA activity (Figure 1).

**Table 1:** Number of laboratory‐confirmed influenza A and B viruses isolated and tested for susceptibility to neuraminidase inhibitors, 2007‐2013, South Africa

	N	2007 n (%)	2008 n (%)	2009 n (%)	2010 n (%)	2011 n (%)	2012 n (%)	2013 n (%)
Influenza A/B	6341	511 (8.1)	389 (6.1)	1753 (27.6)	895 (14.1)	1142 (18.0)	744 (11.7)	907 (14.3)
Influenza subtypes								
A(H1N1)	454	141 (31.0)	309 (68.1)	4 (0.9)	0 (0.0)	0 (0.0)	0 (0.0)	0 (0.0)
A(H1N1)pdm09	2414	—	—	712 (29.5)	211 (8.7)	882 (36.5)	6 (0.2)	603 (25.0)
A(H3N2)	2079	209 (10.1)	13 (6.3)	865 (41.6)	237 (11.4)	159 (7.6)	439 (21.1)	157 (7.6)
aA no subtype	130	27 (20.8)	16 (12.3)	59 (45.4)	1 (0.8)	14 (10.8)	10 (7.7)	3 (2.3)
B	1373	134 (9.8)	51 (3.7)	122 (8.9)	468 (34.1)	128 (9.3)	318 (23.2)	152 (11.1)
NA enzyme inhibition	307							
A(H1N1)	86	39 (45.3)	47 (54.7)	0 (0.0)	—	—	—	—
A(H1N1)pdm09	105	—	—	39 (37.1)	27 (25.7)	26 (24.8)	0 (0.0)	13 (12.4)
A(H3N2)	21	1 (4.8)	0 (0.0)	0 (0.0)	9 (42.9)	2 (9.5)	7 (33.3)	2 (9.5)
B	95	24 (25.3)	24 (25.3)	9 (9.5)	4 (4.2)	20 (21.1)	7 (7.4)	7 (7.4)

N, total number; NA, neuraminidase; (%) = n/N × 100 for each row; —, means relevant subtype did not circulate.

a Subtype or serotype could not be determined.

### 3.2 Neuraminidase inhibitor susceptibility of influenza A and B isolates

A total of 307 influenza virus isolates from 2007 to 2013 were tested for susceptibility to NAIs in the NA enzyme inhibition assay, including 135 influenza viruses isolated during 2007‐2008 (Table 1). Thirty‐nine influenza A(H1N1) isolates from 2007 were susceptible to oseltamivir with median IC_50_: 0.08 nmol/L (range 0.01‐3.60) and to zanamivir with median IC_50_: 0.34 nmol/L (range 0.33‐12.60) (Table 2). Four virus isolates were outliers: 2/4 for oseltamivir and 3/4 for zanamivir.

**Table 2:** Susceptibility to neuraminidase inhibitors of influenza A and B viruses isolated during 2007‐2013 in South Africa

2007‐2008			Oseltamivir, IC_50_ in nmol/L					Zanamivir, IC_50_ in nmol/L				
Influenza type	Year	Isolates tested (N)	Median (range)	IQR	X_0.75_	Statistical cutoff (*X*_0.75_ + 3IQR)	Susceptible	Median (range)	IQR	*X*_0.75_	Statistical cutoff (*X*_0.75_ + 3IQR)	Susceptible
*A(H1N1)*	*2007*	*39*	*0.08 (0.01‐3.60)*	*0.61*	*0.64*	*2.47*	*Yes*	*0.34 (0.33‐12.60)*	*0.56*	*0.71*	*2.39*	*Yes*
	*2008*	*47* ^*a*^	*73.00 (1.56‐305)*	*67.18*	*117.6*	*319.14*	*No*	0.39 (0.13‐1.99)	0.09	0.43	0.70	Yes
A(H3N2)	2007‐2008	1	N/A	N/A	N/A	N/A	N/A	N/A	N/A	N/A	N/A	N/A
Influenza B	2007‐2008	48	2.84 (1.76‐5.47)	1.70	3.85	8.95	Yes	1.99 (1.27‐2.89)	1.29	2.20	6.07	Yes

2009‐2013			Oseltamivir, IC_50_ in nmol/L					Zanamivir, IC_50_ in nmol/L				
Influenza type	Year	Isolates tested (N)	Median (range)	IQR	*X*_0.75_	Statistical cutoff (*X*_0.75_ + 3IQR)	Susceptible	Median (range)	IQR	*X*_0.75_	Statistical cutoff (*X*_0.75_ + 3IQR)	Susceptible
A(H1N1)pdm09	2009‐2013	105^b^	0.11 (0.01‐0.78)	0.14	0.18	0.60	Yes	0.35 (0.27‐0.533)	0.07	0.40	0.61	Yes
A(H3N2)	2009‐2013	20^c^	0.05 (0.01‐0.08)	0.03	0.06	0.15	Yes	0.56 (0.47‐0.66)	0.12	0.63	0.99	Yes
Influenza B	2009‐2013	47^d^	3.20 (1.56‐5.40)	1.47	4.01	8.42	Yes	2.38 (0.91‐5.48)	1.35	3.01	7.06	Yes

IC_50_, 50% inhibition concentration; nmol/L, nanomolar; IQR, interquartile range (=75th percentile‐25th percentile); *X*_0.75_, 75th percentile.

Reasons for discrepant number of isolates assayed for oseltamivir vs zanamivir: ^a^H1N1 (zanamivir): 25/47 tested, 22 insufficient specimens; ^b^H1N1pdm09: 103/105, two failed for both drugs; zanamivir: 97/103, six failed (one showed no inhibition as IC_50_ could not be calculated, five insufficient); ^c^H3N2 (oseltamivir): 18/20 included, two failed; ^d^B(zanamivir): 46/47 included, one insufficient sample. Italic font: outliers with increased IC_50_ values (>10× median and >statistical cutoff) present in data.

In 2008, the median IC_50_ values for oseltamivir increased markedly (median 73 nmol/L; range 1.56‐305 nmol/L) with 97.9% (46/47) for virus isolates. There was a 912‐fold increase in median IC_50_ values between isolates from 2007 and resistant isolates from 2008. Fifty‐three percent (25/47) of influenza A(H1N1) isolates from 2008 remained susceptible to zanamivir (Table 2). Four sporadic cases of seasonal influenza A(H1N1) were detected in 2009, but NAI susceptibility was not determined (Table 1).

During 2007‐2008, all except 1/6 A(H3N2) isolates had low NA activity. IC_50_ for oseltamivir and zanamivir was 0.05 nmol/L and 0.48 nmol/L, respectively, for this isolate. Influenza B isolates were susceptible to both NAIs (Table 2).

For 2009‐2013, 172 influenza virus isolates were tested for susceptibility to NAIs (Table 1). Influenza A(H1N1)pdm09 isolates were susceptible to oseltamivir (98.1%; 103/105) and zanamivir (92.4%; 97/105) with respective median IC_50_ values of 0.11 nmol/L (range 0.01‐0.78) and 0.35 nmol/L (range 0.27‐0.533) (Table 2).Influenza A(H3N2) isolates were all susceptible to inhibition by oseltamivir (n = 18) and zanamivir (n = 20) with median IC_50_ values of 0.05 nmol/L (range 0.01‐0.08) and 0.56 nmol/L (range 0.47‐0.66), respectively during 2009‐2013 (Table 2). Influenza B virus isolates (n = 47) from 2009 to 2013 were susceptible to oseltamivir and zanamivir (Table 2).

### 3.3 Amino acid substitutions in NA genes of influenza viruses

**Figure 2:**
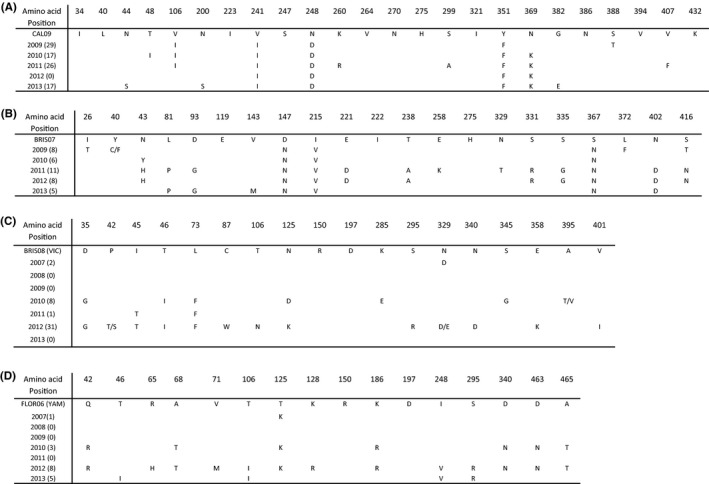
Polymorphism of amino acids in the neuraminidase of (A) influenza A(H1N1)pdm09 viruses aligned with A/California/07/2009 reference (CAL09); (B) influenza A(H3N2) viruses aligned with A/Brisbane/10/07 reference (BRIS07); (C) influenza B viruses of Victoria lineage (VIC) aligned with B/Brisbane/60/2008 reference (BRIS08); and (D) influenza B viruses of Yamagata (YAM) lineage aligned with B/Florida/2006 reference (FLOR06), 2009‐2013, South Africa. Amino acid is designated using standard one letter code. Numbers in parenthesis indicate the number of viruses’ sequences included in analysis

No seasonal influenza A(H1N1) viruses from 2007 to 2008 were sequenced. A total of 140 partial or near‐complete NA gene sequences were generated (43 A(H1N1)pdm09, 38 A(H3N2), 42 B/Victoria, and 17 B/Yamagata lineage) for 2009‐2013. Deduced NA amino acid sequences showed the absence of the oseltamivir resistance mutation H275Y in influenza A(H1N1)pdm09 isolates compared to A/California/07/2009 as reference (Figure 2a). Amino acid mutations were observed at positions 44, 48, 106, 200, 241, 248, 260, 299, 351, 369, 382, and 407 when compared to the reference strain, A/California/07/2009. Some amino acid mutations became fixed: V241I, N248D, and Y351F. The N369K mutation first observed in 2010 viruses also became fixed. None of these mutations are associated with reduced susceptibility to NAIs.

Amino acid substitutions associated with continued evolutionary changes in the NA gene of influenza A(H3N2) viruses were observed at positions 26, 40, 43, 81, 93, 143, 147, 215, 221, 238, 258, 329, 331, 335, 367, 372, 402, and 416. The D147N, I215V, S367N mutations became fixed from 2009 to 2013 compared to the A/Brisbane/10/07 reference strain (Figure 2b). These mutations were not associated with resistance to NAIs.

No amino acid substitutions associated with reduced susceptibility to oseltamivir and zanamivir were observed in deduced NA protein sequences of influenza B lineages, specifically at positions 150 and 197 (Figure 2c and 2d). Drift amino acid substitutions were observed at several amino acid positions in 2010‐2012 for B/Victoria and 2010‐2013 for B/Yamagata viruses.

## 4 DISCUSSION

In this study, we confirm change in circulation of oseltamivir‐susceptible seasonal influenza A(H1N1) strains in 2007 to almost 100% resistant phenotype strains in 2008 in South Africa. During the same period, Cameroon reported resistant phenotype strains for 70% of A(H1N1) viruses.^43^ During the same period, a single influenza A(H3N2) and all influenza B virus isolates from our study remained susceptible to both NAIs.

Influenza A(H1N1)pdm09 and A(H3N2) virus isolates from 2009 to 2013 and 2007 to 2013, respectively, also remained susceptible to inhibition by both NAIs despite the observed increased IC_50_ values for some isolates. No resistance was detected for influenza B viruses. Since 2009, seasonal influenza A(H1N1) has not been detected during seasonal epidemics and has been replaced by A(H1N1)pdm09.^44^ Influenza viruses (H1N1 and H1N1pdm09) carrying the H275Y mutation and associated with clinical resistance to oseltamivir displayed > 400‐fold increase in IC_50_.^9^, ^45^

No seasonal influenza A(H1N1) NAs were sequenced in this study. However, in 2008 the H275Y substitution was reported in resistant strains from South Africa.^9^ Circulation of resistant phenotype seasonal influenza A(H1N1) was aborted with emergence of the influenza A(H1N1)pdm09 strain. Since 2009, we did not detect amino acid mutations associated with reduced susceptibility to NAIs. However, the V241I and N369K mutations in resistant influenza A(H1N1)pdm09 isolates are thought to increase the stability of neuraminidases with H275Y substitutions.^46^ The I215V NA substitution was observed in A(H3N2) viruses that circulated in the 2013‐2014 season and caused borderline reduction in susceptibility to oseltamivir and zanamivir.^10^ These observations highlight the need for continued surveillance even if NAIs are not widely used in South Africa.

Resistance to inhibition by oseltamivir was reported at frequencies of 0.2% (2/900 adults) to 1.1% (2/190 children) for influenza A(H3N2) from combined studies including clinical trials conducted in Japan.^47^ Oseltamivir resistance was mostly associated with E119V and R292K mutations.^47^ In global surveillance, data from 2013 to 2014 H3N2‐resistant phenotype isolates were reported at 0.3%.^10^ We observed that most A(H3N2) isolates displayed low NA activity, and therefore, NA susceptibility to antivirals could not be assessed. Our inability to obtain A(H3N2) isolates with high levels of NA activity could be related to changes in phenotypic characteristics of A(H3N2) viruses.^48^, ^49^ During virus isolation, only immunofluorescence and hemagglutination assays were routinely used to detect virus growth, which may further account for low number of isolates subsequently identified with high NA levels.

Despite observed continued drift in B lineages in this study, these mutations were not associated with NAI resistance or reduced susceptibility. The influenza B/Memphis/20/96 strain carrying the R150K mutation associated with highly reduced resistance to both zanamivir and oseltamivir and used in this study as reference resistant strain was initially isolated from an immune‐compromised patient in the United States following zanamivir treatment.^50^, ^51^ Susceptibility to NAIs was determined for influenza virus isolates from 24 patients [2009: 13 A(H1N1)pdm09 and 2 influenza B; 2012: 9 A(H3N2)] enrolled through the severe acute respiratory illness (SARI) surveillance.^18^, ^19^ Influenza isolates from SARI cases were susceptible to the NAIs oseltamivir and zanamivir (data not shown).

Limitations to our study were as follows: (a) No severe cases were included; (b) virus culture supernatant fluids used were retrieved from freezers. Strains were tested at passages 1‐2 to limit mutations introduced through repeated passaging. We are unsure of the effects of long‐term storage and possible freeze‐thawing on NA concentrations but others used inactivated viruses in the IC_50_ assay^27^; and (c) only a small proportion of influenza‐positive cases were tested annually.

This study provides baseline data on influenza A and B viruses with oseltamivir‐susceptible phenotypes in South Africa. Guidelines for the prevention and treatment of influenza in South Africa are published electronically.^52^ However, although oral oseltamivir and inhaled zanamivir are available in South Africa, they are not routinely prescribed.^14^, ^53^ This is also observed for hospitalized patients enrolled through our pneumonia surveillance program in public hospitals (C. Cohen, unpublished data). Oseltamivir is prescribed more frequently by private healthcare practitioners, but no data are available on the extent of use in South Africa. Given the very low frequency of resistance reported globally, the use of NAIs to treat patients at high risk of developing severe illness should be promoted.

## CONFLICT OF INTEREST

The authors do not declare financial or personal conflict of interest related to this study.

## DISCLAIMER

The findings and conclusions in this report do not necessarily represent the official position of the US Centers for Disease Control and Prevention. The corresponding author has full access to all data and final responsibility for the decision to submit this publication.
